# Analysis of the EIAV Rev-Responsive Element (RRE) Reveals a Conserved RNA Motif Required for High Affinity Rev Binding in Both HIV-1 and EIAV

**DOI:** 10.1371/journal.pone.0002272

**Published:** 2008-06-04

**Authors:** Jae-Hyung Lee, Gloria Culver, Susan Carpenter, Drena Dobbs

**Affiliations:** 1 Bioinformatics and Computational Biology Program, Department of Genetics, Development and Cell Biology, Iowa State University, Ames, Iowa, United States of America; 2 Department of Biology, University of Rochester, Rochester, New York, United States of America; 3 Department of Veterinary Microbiology and Pathology, Washington State University, Pullman, Washington, United States of America; University of Hong Kong, China

## Abstract

A cis-acting RNA regulatory element, the Rev-responsive element (RRE), has essential roles in replication of lentiviruses, including human immunodeficiency virus (HIV-1) and equine infection anemia virus (EIAV). The RRE binds the viral trans-acting regulatory protein, Rev, to mediate nucleocytoplasmic transport of incompletely spliced mRNAs encoding viral structural genes and genomic RNA. Because of its potential as a clinical target, RRE-Rev interactions have been well studied in HIV-1; however, detailed molecular structures of Rev-RRE complexes in other lentiviruses are still lacking. In this study, we investigate the secondary structure of the EIAV RRE and interrogate regulatory protein-RNA interactions in EIAV Rev-RRE complexes. Computational prediction and detailed chemical probing and footprinting experiments were used to determine the RNA secondary structure of EIAV RRE-1, a 555 nt region that provides RRE function in vivo. Chemical probing experiments confirmed the presence of several predicted loop and stem-loop structures, which are conserved among 140 EIAV sequence variants. Footprinting experiments revealed that Rev binding induces significant structural rearrangement in two conserved domains characterized by stable stem-loop structures. Rev binding region-1 (RBR-1) corresponds to a genetically-defined Rev binding region that overlaps exon 1 of the EIAV rev gene and contains an exonic splicing enhancer (ESE). RBR-2, characterized for the first time in this study, is required for high affinity binding of EIAV Rev to the RRE. RBR-2 contains an RNA structural motif that is also found within the high affinity Rev binding site in HIV-1 (stem-loop IIB), and within or near mapped RRE regions of four additional lentiviruses. The powerful integration of computational and experimental approaches in this study has generated a validated RNA secondary structure for the EIAV RRE and provided provocative evidence that high affinity Rev binding sites of HIV-1 and EIAV share a conserved RNA structural motif. The presence of this motif in phylogenetically divergent lentiviruses suggests that it may play a role in highly conserved interactions that could be targeted in novel anti-lentiviral therapies.

## Introduction

Retroviruses, including the lentiviruses HIV-1 and EIAV, employ a variety of mechanisms to regulate the expression of alternatively spliced viral mRNAs [1], [2]. The presence of suboptimal splice sites allows for differential expression of several mRNAs from a single pre-RNA. Regulatory *cis*-acting RNA sequences that either enhance or repress recognition of a splice site by the host cellular splicing factors often play critical roles in regulating splice-site selection [3], [4]. In some retroviruses, viral pre-mRNAs contain constitutive transport elements (CTE) that are recognized by cellular proteins to facilitate nuclear export of incompletely spliced viral mRNAs [5]. Other retroviruses encode Rev/Rex proteins that act in *trans* to regulate nuclear export of unspliced or incompletely spliced mRNAs required for expression of structural and enzymatic proteins as well as progeny viral RNA genomes [6]–[8].

The Rev/Rex RNA export pathway has been best characterized in HIV-1 [9]. After entering the nucleus, the HIV-1 Rev protein binds to a specific *cis*-acting element, termed the Rev-responsive element (RRE), within the viral pre-mRNA [10], [11], multimerizes [12], [13] and then facilitates nuclear export of incompletely spliced viral mRNA via the Crm1 nuclear export pathway [14], [15]. Discrete functional domains within Rev mediate nuclear localization, RRE binding and multimerization, and nuclear export. The HIV-1 RRE is a highly structured RNA located at the junction between the SU (gp120) and TM (gp41) domains of the *env* gene [11], [16], [17]. Biochemical and biophysical experiments have implicated specific stem-loop structures in Rev binding and multimerization [18]–[20]. Because the Rev-dependent export pathway plays an essential role in HIV-1 replication, disruption of the Rev-RRE interactions is an attractive target for design of effective antiviral therapies [21], [22]

All lentiviruses utilize the Rev-dependent, Crm1-mediated export pathway for expression of incompletely spiced mRNAs. There is no conservation among the lentiviral RREs at the RNA sequence level; however, the RRE regions of several primate and non-primate lentiviral genomes map to the SU/TM junction in *env* gene [11], [16], [17], [23], [24]. In addition, computational analyses have suggested that several of the lentiviral RREs may share RNA secondary structural elements [25]. Equine infectious anemia virus (EIAV) is one of the most divergent members of the subfamily [26]. EIAV Rev (ERev) is functionally homologous with other lentivirus Revs and utilizes the Crm1 pathway for export of incompletely spliced mRNAs, yet EIAV differs from most lentiviruses in structural and functional features of Rev and RRE. EIAV therefore offers an opportunity for comparative analysis of the molecular interactions important in regulation of lentiviral gene expression.

**Figure 1:**
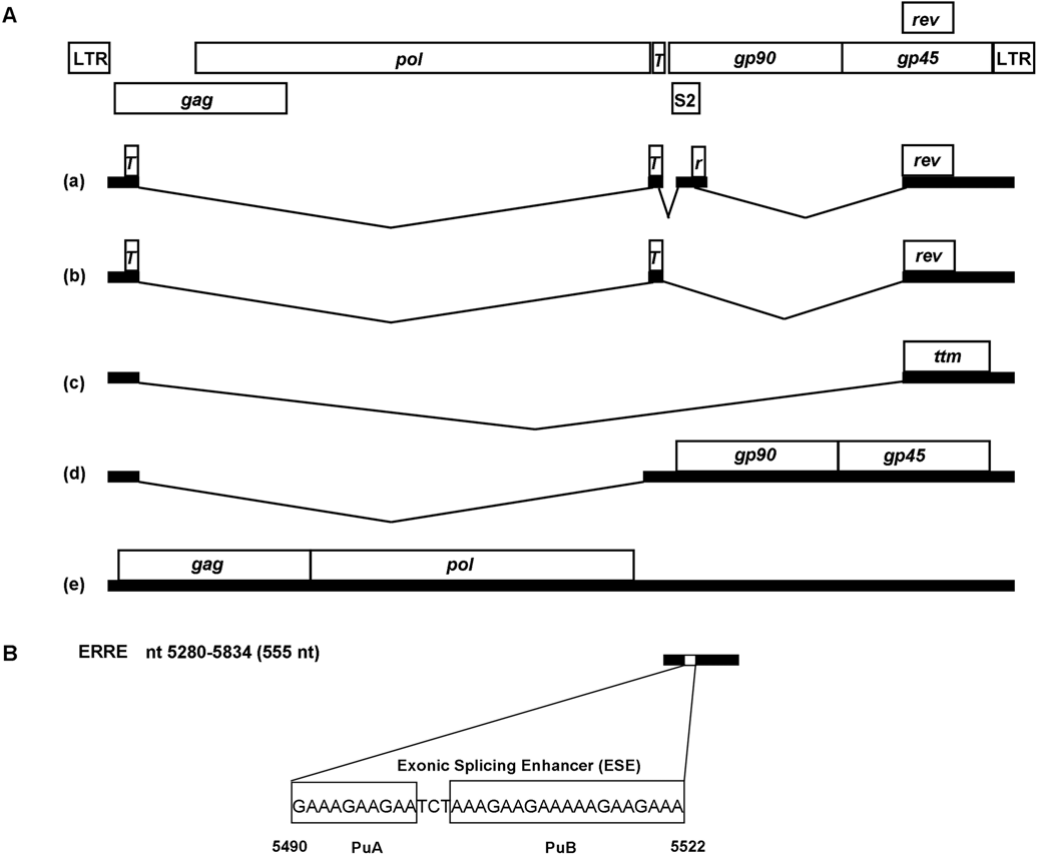
Organization of EIAV genome, transcript splicing patterns, and location of the ERRE sequence. (A) Schematic view of the EIAV genome showing the locations of open reading frames and alternatively spliced mRNA transcripts generated from the EIAV genome: mRNA transcript (a) encodes both the Tat (T) and Rev (r, rev) proteins. In the presence of Rev protein, EIAV exon 3 is skipped and the Tat (T) protein is produced from mRNA (b). mRNA (c) encodes Ttm, a protein of unknown function. Structural and enzymatic proteins are translated from mRNAs (d) and (e). Unspliced mRNA (e) corresponds to progeny RNA that is packaged to produce infectious virus. (B) Sequence of the exonic splicing enhancer (ESE) within the 555 nt ERRE (genomic location, nt 5280–5834), previously designated as ERRE-1 [34]. Boxed sequences represent two purine-rich sequence stretches (PuA and PuB) previously reported to interact with both the EIAV Rev protein and host protein SF2-ASF [29], [32].

EIAV Rev is a 165 amino acid protein translated from exons 3 and 4 of a multiply spliced, four-exon, bicistronic mRNA that also encodes the *trans*-activating protein, Tat (Figure 1). The leucine-rich nuclear export signal (NES) in ERev is similar to other viral and cellular export proteins that interact with the Crm1, but is atypical in the spacing of the leucine residues within the NES [14]. The ERev RNA-binding domain is bipartite, comprising two short arginine-rich motifs (ARMs) separated by 79 amino acids in the primary sequence [27]. It is not clear how the two domains cooperate to form a complex with the RRE, but a theoretical structural model of the ERev protein places the ARMs in close proximity within the three-dimensional structure, suggesting they could form a single RNA binding interface within the Rev-RRE complex [27] (Lee and Ihm, personal communication). In addition to promoting nuclear export of incompletely spliced RNA, ERev also regulates inclusion of exon 3 in the multiply spliced bicistronic mRNA: in the presence of Rev, exon 3 is skipped, resulting in a three-exon, monocistronic mRNA encoding only Tat [28], [29]. Exon 3 is flanked by a suboptimal splice acceptor and contains a purine-rich, exonic splicing enhancer (ESE) required for exon 3 inclusion [30]. ESEs are typically purine-rich sequences, embedded within alternatively spliced exons, which bind cellular SR proteins and recruit essential splicing factors to suboptimal splice sites, resulting in inclusion of alternatively spliced exons. It is thought that Rev-mediated skipping of exon 3 is a consequence of Rev interacting either with SR proteins, or with ESE RNA (or with both) to disrupt ESE-SR protein interactions [29]–[32].

The EIAV RRE (ERRE) differs from other lentiviral RREs with respect to location and function. The ERRE is located in a 555 nt region near the 5′ end of *env*, which spans exon 3 of the Tat-Rev mRNA [29], [31], [33], [34]. A 57 nt sequence encompassing the ESE within exon 3 was shown to bind GST-Rev and to act as a functional RRE in a heterologous nuclear export assay system [29]; however, nuclear export activity of the 57 nt “minimal ERRE” was reduced compared to the full-length ERRE [34]. Mutational analyses of the 555 nt ERRE demonstrated that purine-rich sequences within exon 3 function as both an ESE and an RRE [30], [32], [34]. The ERRE thus plays an important role in the complex interactions between viral pre-mRNAs, the viral Rev protein, and cellular splicing factors, to mediate alternative splicing and regulation of viral gene expression [30]–[32], [35].

In this paper, we investigate the RNA structure of the EIAV RRE and its interactions with the Rev protein. We propose an RNA secondary structure model for the essential RRE in EIAV, based on a combination of secondary structure prediction and chemical probing experiments. We present detailed *in vitro* footprinting analysis of EIAV Rev-RRE complexes, and identify two distinct domains within the essential RRE that undergo significant structural transitions upon Rev binding. Computational analyses identified an RNA secondary structural motif within the high affinity Rev-binding sites of both HIV-1 and EIAV that is present within the mapped RREs of four additional lentiviruses. The discovery of a conserved recognition element for lentiviral Rev-RRE interactions lays the groundwork for investigating highly conserved RNA-protein interactions that could be targeted in novel anti-lentiviral therapies.

## Results

### Probing the RNA secondary structure of the ERRE

**Figure 2:**
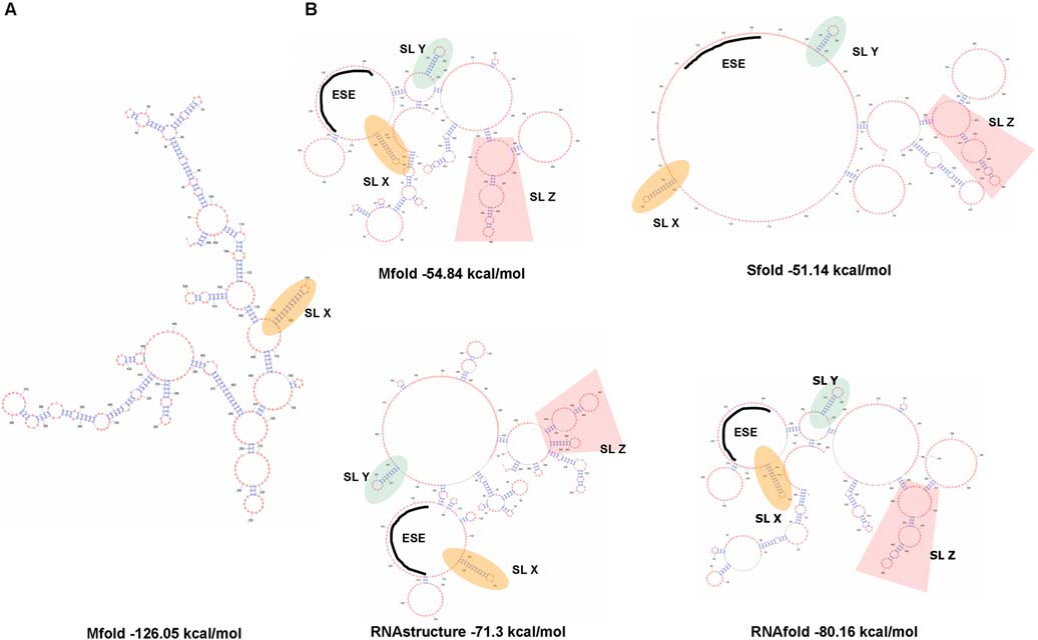
RNA secondary structural models for the ERRE. (A) The lowest free energy secondary structure of the ERRE, predicted by Mfold without incorporatating experimental constraints [36]. (B) Lowest free energy RNA secondary structure models for the ERRE, generated by 4 different algorithms, all using chemical probing results as experimental constraint input: Mfold, Sfold [38], RNAStructure [39], and RNAfold [40]. SL-X, -Y and -Z, stem-loop structures common to all four models, are highlighted; the exonic splicing enhancer (ESE) is indicated by heavy lines (see Materials and Methods for details).

The secondary structure of the ERRE was analyzed using a combination of computational and experimental approaches. Figure 2A shows the lowest free energy structure of the ERRE predicted using Mfold [36] with default parameters. We experimentally interrogated the secondary structure by analyzing the accessibility of ribonucleotides in the folded RNA to single-strand specific chemical probes (kethoxal and DMS), thus identifying regions not involved in base-pairing [37]. Chemically-modified nucleotides in the ERRE were identified by primer extension analysis using 6 different primers (Table 1). Experimental data from chemical probing experiments were integrated into computational predictions of secondary structure of the ERRE, using several different algorithms designed to incorporate experimental constraints from chemical probing assays. Figure 2B illustrates four different secondary structure models generated by Mfold [36], Sfold [38], RNAstructure [39], and RNAfold [40] using our experimental constraints. Although the latter four models differ in detail, they are similar in overall topology: all four models share a set of 3 stem-loop structures (SL-X, -Y, and -Z) and in every case, the ESE is located within a large loop (heavy line in Figure 2B). Notably, with the exception of SL-X, none of these shared features are also found in the structure generated without experimental constraints (Figure 2A).

**Table 1:** Oligonucleotide primers used in the primer extension analysis of ERRE

Primers	Nucleotide sequence
Primer 90	5′- TTTTTCTGACTGTTGGG -3′
Primer 185	5′- TCTTGGTCTCTTGCTTC -3′
Primer 291	5′- CCAAAGTATTCCTCCAG -3′
Primer 389	5′- CCCCAGCATTCTATAGC -3′
Primer 485	5′- GCTTCTAATAATGTAGC -3′
Primer 555	5′- TCCCCAATATTCCGCTGTGT -3′

Numbers in primer names refer to the position of the 5′ end of each primer, relative to the 5′ end of ERRE-1 (see Materials and Methods for details.)

**Figure 3:**
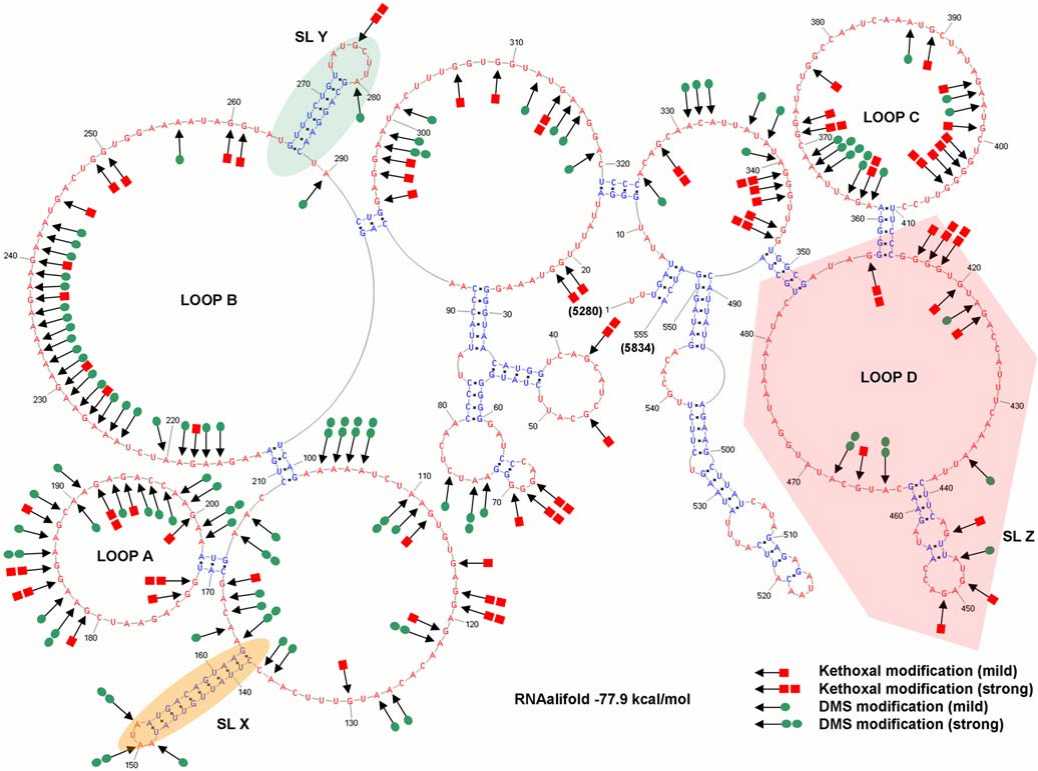
Chemical probing results mapped onto the RNA secondary structure of ERRE. RNAalifold [41] was used to generate an optimized RNA secondary structure of the ERRE based on a combination of thermodynamic considerations, experimental constraints, and sequence covariation information derived from multiple sequence alignment of a collection of 140 ERRE sequence variants. Arrowheads denote ribonucleotides modified by chemical probing reagents: kethoxal (red squares) and DMS (green circles), with the relative extent of modification represented by either two (strong) or one (weak) symbol. SL-X, -Y and -Z are stem-loop structures also shown in Figure 2.

To further refine and validate the RRE secondary structure models, we used available sequence information from EIAV variants to perform covariation analyses, using RNAalifold [41]. RNAalifold can incorporate covariation information from a collection of aligned RNA sequences, in addition to experimental constraint data, as input. Thus, the algorithm determines a consensus RNA secondary structure based on thermodynamic considerations and then evaluates the compatibility of observed sequence covariations with that secondary structure. Experimental constraints are used in the final step to identify an optimized RNA secondary structure. A total of 140 different EIAV SU variant sequences [42] that overlap the ERRE were used to generate a multiple sequence alignment for computing ribonucleotide covariation frequencies within the ERRE. The secondary structure generated by RNAalifold is shown in Figure 3. Arrows indicate ribonucleotides for which chemical probing experiments revealed accessibility to modification by kethoxal (red rectangles) or DMS (green circles). The number of rectangles or circles corresponds to the intensity of the cleavage band at that position, with more symbols indicating a higher probability of “single-strandedness.” The overall topology of this secondary structure of the ERRE is very similar to the secondary structures presented in Figure 2. In the optimized model shown in Figure 3, 373 of 555 ribonucleotides in the ERRE participate in the base-pairing and 182 are located in single-stranded regions. The estimated free energy for this structure, based on a combination of thermodynamic considerations, chemical probing results and covariation analyses, is −77.90 kcal/mol. Notably, the ESE and the previously identified EIAV Rev binding region [27], [29], [32], are both located within the single-stranded loop B. Several structural features, including loop B and stem-loop regions SL-X and SL-Y, are the same in all five models shown in Figure 2B and Figure 3. The inclusion of covariation information results in one significant difference: in SL-Z, a short stem formed by base-pairing between nt 425–428 and nt 470–473, which connects two small internal loops (Figure 2B), is converted to a single-stranded region in the optimized RNA secondary structure model shown in Figure 3, creating a single larger loop (loop D).

### EIAV Rev “footprints” two regions within the ERRE

Previous experiments had implicated the purine-rich ESE within the ERRE as a primary binding site for EIAV Rev protein [29], [32]. To obtain detailed information regarding the interaction of EIAV Rev with ERRE sequences and structural motifs, we performed RNA “footprinting” experiments, using chemical probing and primer extension analysis [43], [44]. Three different chemical reagents were used to compare the accessibilities of ribonucleotides in the ERRE to modification in the presence or absence of bound ERev. Hydroxyl radicals were used to monitor cleavages in the sugar-phosphate backbone, and kethoxal (which modifies N1 and N2 of guanines) and DMS (which methylates N1 of adenines and N3 of cytosines) were employed as base-specific probes. The folded ERRE RNA alone or preformed ERev-ERRE complexes were subjected to modification by each chemical reagent. Primer extension by reverse transcriptase was used to identify the chemically-modified ribonucleotides in the ERRE sequence. In ladders of ^32^P-labeled primer extension products visualized by denaturing polyacrylamide gel electrophoresis, sites of modification correspond to a “stop” or an enhanced band located one position downstream (3′) of the band corresponding to the modified ribonucleotide site.

**Figure 4:**
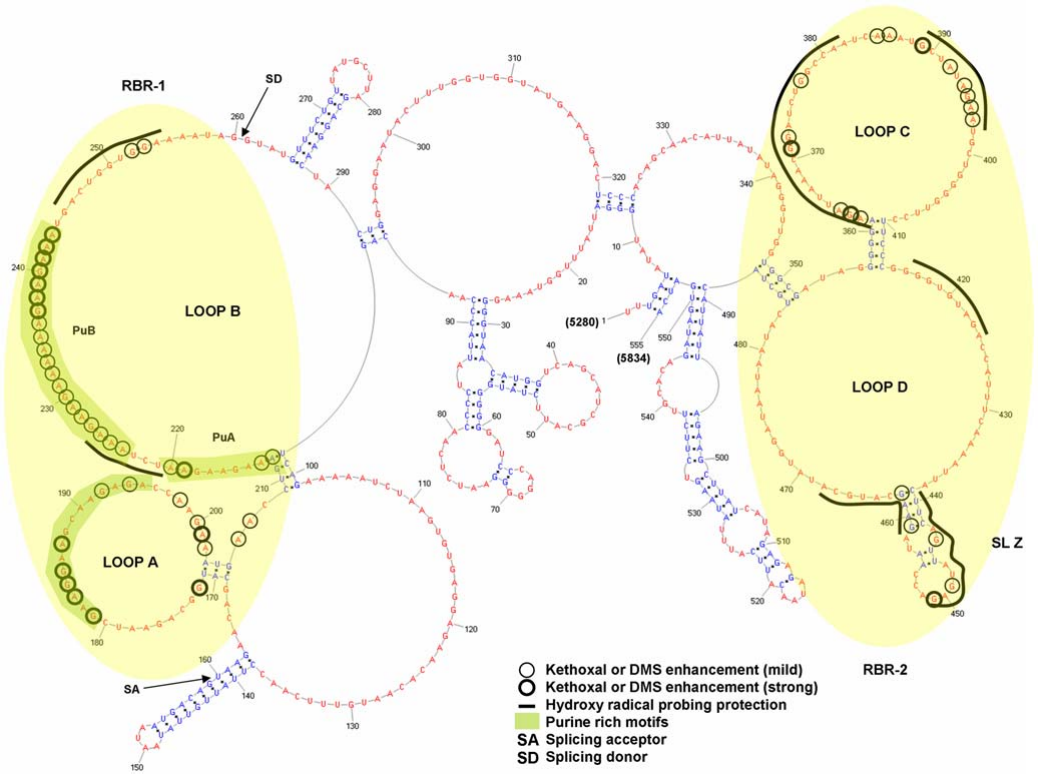
Two distinct regions of the ERRE undergo structural transitions in the presence of bound EIAV Rev protein. Consensus chemical modification patterns, based on at least 3 experiments in which several different primers were used to probe the complete ERRE RNA, mapped onto the RNA secondary structure model shown in Figure 3. Ribonucleotides that were consistently displayed enhanced modification with either kethoxal or DMS upon Rev binding are circled: bold circle (strong) and thin circle (mild). Regions protected from hydroxyl radical cleavage in the presence of Rev are denoted by a thick line. Purine-rich motifs are highlighted in green.

**Figure 5:**
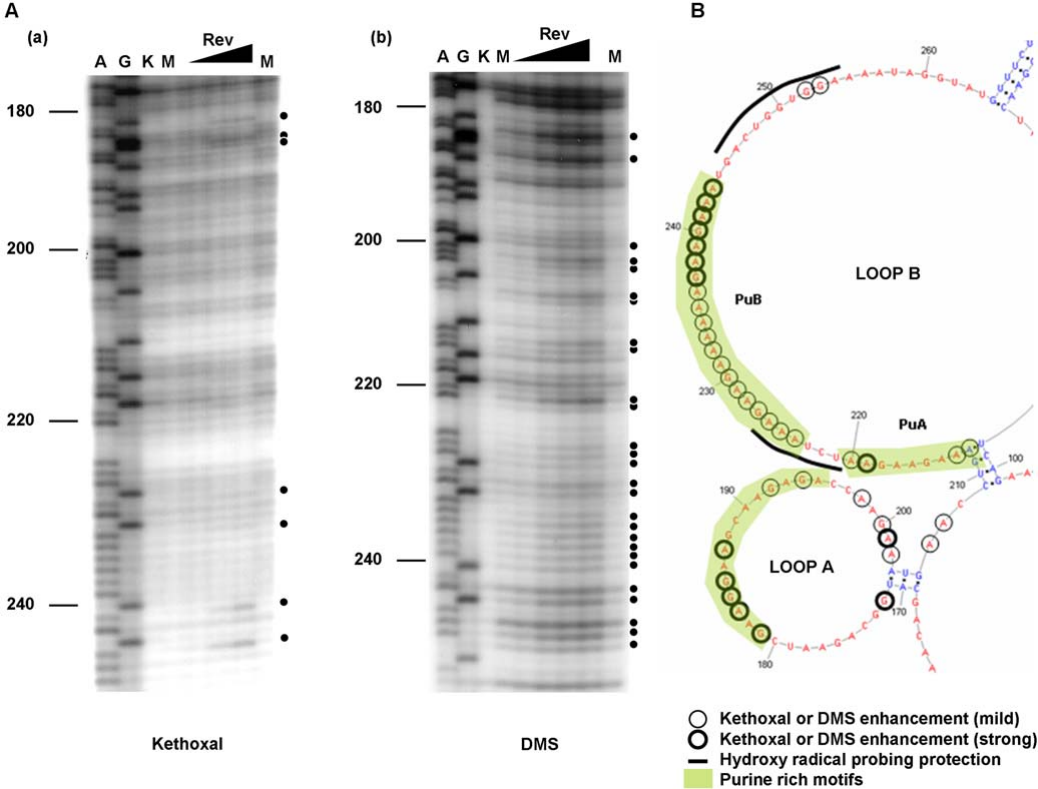
Representative footprinting results for RBR-1. (nt 5449–5539, genomic location). (A) Representative gels from primer extension analysis of chemical probing experiments using kethoxal (a) and DMS (b). Similar experiments were performed using the hydroxyl radical cleavage reagent, Fe-EDTA (data not shown). Circled ribonucleotides denote positions with enhanced reactivity in the presence of bound EIAV Rev protein (“footprints”). Lanes A & G, Dideoxy sequencing markers; lane K, control, unmodified ERRE, in the absence of Rev; lane M, ERRE modified in the absence of Rev; (lanes ▴) ERRE modified in the presence of increasing amounts of Rev protein (1∼30 fold molar excess). (B) Consensus chemical modification patterns in RBR-1, based on several experiments similar to those illustrated in part (A), are mapped onto the corresponding portion of the RNA secondary structure of the ERRE (from Figure 3). Ribonucleotides that consistently display enhanced modification with either kethoxal or DMS upon Rev binding are circled: bold circle (strong) and thin circle (mild). Regions protected from hydroxyl radical cleavage in the presence of Rev are denoted by a thick line. Purine-rich motifs are highlighted in green.

**Figure 6:**
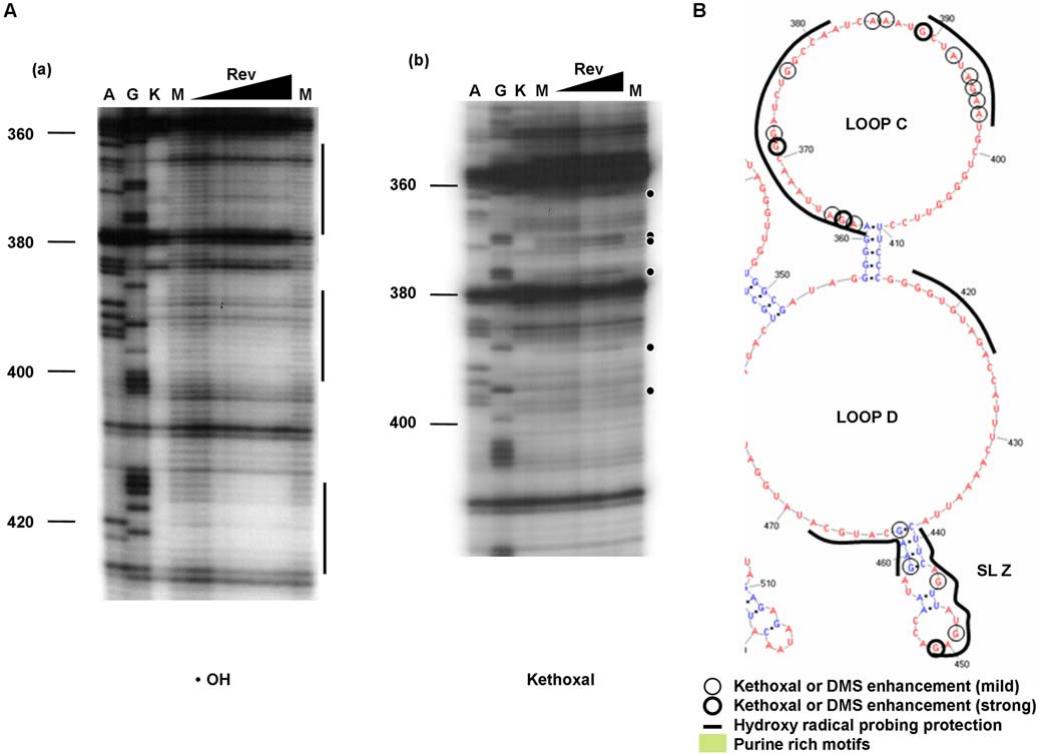
Representative Rev footprinting results in RBR-2. (nt 5639–5749, genomic location). (A) Representative gels from primer extension analysis of chemical probing experiments using hydroxyl radical cleavage reagent, Fe-EDTA (•OH) (a) and DMS (b). Similar experiments were performed using kethoxal (data not shown). Circled ribonucleotides denote positions with enhanced reactivity in the presence of bound EIAV Rev protein (“footprints”). Lanes A & G, Dideoxy sequencing markers; lane K, control, unmodified ERRE, in the absence of Rev; lane M, ERRE modified in the absence of Rev; (lanes ▴) ERRE modified in the presence of increasing amounts of Rev protein (1∼30 fold molar excess). (B) Consensus chemical modification patterns in RBR-2, based on several experiments similar to those illustrated in part (A), are mapped onto the corresponding portion of the RNA secondary structure of the ERRE (from Figure 3). Ribonucleotides that consistently display enhanced modification with either kethoxal or DMS upon Rev binding are circled: bold circle (strong) and thin circle (mild). Regions protected from hydroxyl radical cleavage in the presence of Rev are denoted by a thick line. Purine-rich motifs are highlighted in green.

A summary of the footprinting results is shown in Figure 4, and representative footprinting gels are shown in Figures 5 and 6. Two distinct regions within ERRE are “footprinted” by Rev (highlighted in yellow, Figure 4). Rev binding region-1 (RBR-1) is ∼90 nt long (nt 170–260) and encompasses the ESE, including both purine-rich regions PuA and PuB [29], [32]. A second domain, RBR-2, is ∼110 nucleotides long (nt 360–470) and represents a newly identified Rev interaction domain. Positions with enhanced kethoxal and DMS reactivity (circled residues) in ERev-ERRE complexes, compared with the unbound RNA, are located primarily in single-stranded regions of the ERRE secondary structure. Most regions protected from hydroxyl radical cleavage are also in single-stranded loops. The experiments illustrated in Figures 5 and 6, together with many similar experiments using different primers to probe the entire ERRE sequence (Table 1), were used to generate Figure 4, which summarizes the reproducible patterns of significant differences in chemical reactivity of the ERRE upon Rev binding.

### RBR-1: Rev binding induces structural changes both within the ESE in ERRE and in adjacent single-stranded regions

Within RBR-1, corresponding to the 5′ terminal region of the ERRE, several purine-rich regions and GAR (guanine-adenine-purine) motifs have been identified as binding sites for the host splicing factor, SF2/ASF [31], [45]. The purine-rich regions PuA and PuB, in particular, have been shown to bind both EIAV Rev and SF2/ASF in electrophoretic mobility shift assays [27], [29], [31], [32]. To obtain more detailed footprinting information for this region, we systematically analyzed changes in the relative extent of ribonucleotide modification using titration experiments, in which ERev-ERRE complexes were formed with increasing amounts of purified EIAV Rev protein (Figure 5A).

Within loop B, numerous changes in ribonucleotide accessibilities were detected as increasing amounts of EIAV Rev bound, especially in the PuA and PuB regions (Figure 5). In the PuA, enhanced reactivity was seen for 4 out of 9 nucleotides and in PuB, all 20 purine residues showed enhanced reactivity, with both kethoxal and DMS (Figure 5). Two regions of Rev-mediated protection from hydroxyl radical cleavage, which are not as strong as ones in loop C and D (see below), were also observed in loop B, one located in the sequence between PuA and PuB and another immediately downstream from PuB (nt 245–255, Figure 5B). In addition to the two purine-rich motifs within the ESE, a third purine-rich motif, located in loop A, was strongly affected by Rev binding. Enhanced reactivity was seen for 7 out of 15 nucleotides within this motif and for 4 additional purine residues near the stem at the base of loop A (Figure 5B)

Although the region corresponding to loop A, formed by nt 172 to 202, had not previously been implicated in EIAV Rev binding, our footprinting analyses revealed significantly enhanced reactivities of ribonucleotides in this loop. Therefore, we conclude that within RBR-1, in addition to a region within loop B that encompasses previously identified purine-rich PuA and PuB motifs in the ESE, a purine-rich motif in within loop A also undergoes significant structural rearrangement upon Rev binding.

### RBR-2: A newly identified Rev-binding region in ERRE corresponds to a high affinity binding site

By monitoring nucleotide accessibility changes in response to Rev binding across the entire ERRE sequence, we were able to identify RBR-2, a region that had not been previously implicated in Rev binding, located approximately 100 nt downstream from RBR-1. Hydroxyl radical probing identified several regions within loops C and D that are strongly protected from hydroxyl radical cleavage upon EIAV Rev binding (Figure 6). Enhanced DMS and kethoxal reactivities were also observed for several positions within loops C and D. Within RBR-2, most ribonucleotides involved in EIAV Rev binding are located within these large single-stranded regions. However, one stem-loop region, SL-Z, is strongly footprinted by Rev. SL-Z contains two protected stretches (nts 439–451 and 459–468) and several enhanced nucleotide reactivities (e.g., nts 451 and 459) (Figure 6B).

The discovery of interactions between EIAV Rev and RBR-2 is consistent with previous reports that the complete 555 nt ERRE has more functional activity *in vivo* than several shorter ERRE-derived constructs that encompass the ESE region but lack RBR-2 [29]. We hypothesize that Rev-binding sites within RBR-2 are, in fact, the functional elements “missing” in shorter constructs that retain less activity than the complete ERRE *in vivo*. Direct *in vitro* binding affinity measurements support this interpretation: purified ERev binds RNAs that contain both RBR-1 and RBR-2 with an estimated K_d_ = 19 nM, whereas RNAs that lack RBR-2, but still contain RBR-1, have ∼25-fold lower affinity, K_d_ = 5.2 µM (Figure S1, Supporting Information), indicating that RBR-2 is required for high affinity binding of EIAV Rev to the ERRE.

### Conservation of ERRE sequences suggests preservation of RNA structural elements

**Figure 7:**
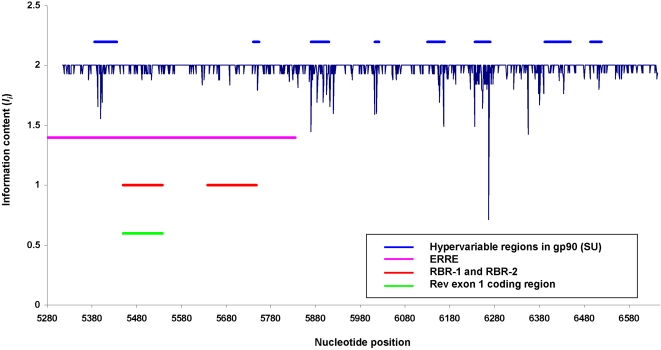
Conservation of RNA sequences in the gp90 (SU) gene of EIAV. Conservation of RNA sequences in EIAV *env* gene (gp90) was assessed by evaluating information content at each nucleotide position in a CLUSTALW-generated multiple sequence alignment of 140 gp90 sequence variants (see Materials and Methods for details). Information content (I) is plotted against nucleotide position, numbered from the first ribonucleotide in the ERRE. The gp90 gene begins at position 35. The maximum information content value is 2, which corresponds to 100% conservation at a particular position in this alignment. The locations of gp90 hypervariable regions, identified in a previous analysis of SU variants [42], are indicated by horizontal blue bars above the graph. Horizontal bars indicate the locations of the ESE (pink), EIAV Rev binding regions 1 and 2 (RBR-1 & RBR-2) (red), and EIAV Rev exon 1 (green).

An analysis of 178 HIV-1 variant sequences revealed that RNA sequences corresponding to the RRE are highly conserved, most likely as a result of evolutionary pressure for maintenance of both the protein sequence encoded by the gp160 gene (within which the HIV-1 RRE is embedded) and the RNA secondary structure of the RRE [46]. To investigate potential selective pressure for maintaining RNA structural elements within EIAV ERRE sequences, we performed information content analyses using a group of variant EIAV sequences. Information content analysis is widely used to evaluate sequence conservation in nucleic acid or protein sequences (e.g., [46]–[49]). Sequences of the EIAV gp90 gene, collected for analysis of envelope SU protein variants by Mealey, et al. [42], were aligned and information content (Shannon entropy) was calculated as described in Materials and Methods. Figure 7 shows the distribution of information content values for ribonucleotide positions in the gp90 gene. The highest possible value for information content is 2, corresponding to cases in which all sequences in the alignment are identical at a particular position. Blue horizontal bars above the plot indicate the locations of previously described hypervariable regions in gp90 [42]; horizontal bars indicating the locations of the ERRE (pink), the Rev exon 1 coding region (green) [2], and the Rev-footprinted regions determined in this study (RBR-1 and RBR-2, red) are also shown. This analysis reveals that, except for one segment corresponding to a known hypervariable region of gp90, the entire ERRE region is highly conserved relative to the rest of the gp90 gene. Notably, both RBR-1 and RBR-2 are located within the most highly conserved region and display similar levels of sequence entropy. Taken together, these results suggest that the observed pattern of sequence conservation in gp90 results from evolutionary pressure to preserve not only protein sequences encoded by the gp90 gene, but also RNA sequence and secondary structural features of the embedded regulatory ERRE region that are required for Rev binding.

### RNA structural motifs found within the high affinity Rev-binding sites of HIV-1 and EIAV are also found in other lentiviral RREs

**Figure 8:**
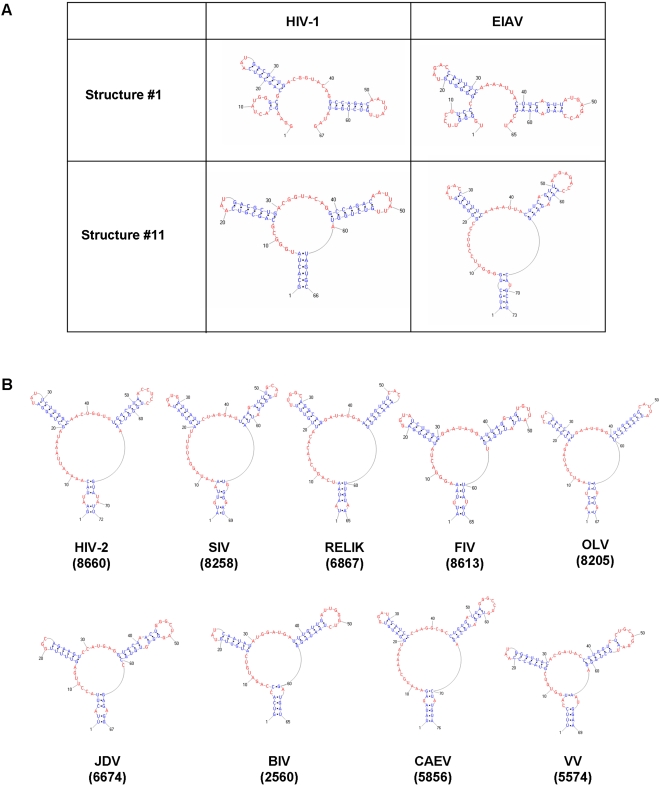
A conserved RNA structural motif identified in the high affinity Rev-binding sites of HIV-1 and EIAV is found within the RREs or *env* genes of diverse lentiviruses. (A) Two representative structures (#1 and #11) from an ensemble of 20 pairs of similar structures are shown. These motifs were identified in 100 nt regions containing high affinity Rev binding sites from HIV-1 or EIAV, which were extracted and structurally aligned using Dynalign [51] to generate ensembles of structures. (B) Similar motifs were identified in genomic RNA sequences of 8 additional lentiviruses. The RNA structural motifs shown in (A) were used to scan the complete genomic RNA sequences of additional lentiviruses: HIV-2, SIV (simian immunodeficiency virus), RELIK (rabbit endogenous lentivirus type K), FIV (feline immunodeficiency virus), OLV (ovine lentivirus), JDV (jembrana disease virus), BIV (bovine immunodeficiency virus), CAEV (caprine arthritis-encephalitis virus), and Visna (visna virus). The motifs shown are examples of those similar to structure #11 in Figure 8A (see Materials and Methods and Table S1 for details).

To explore the possibility that RNA secondary structural elements required for Rev binding in EIAV might also be found in other lentiviruses, we first asked whether RBR-1 and RBR-2, identified in this study, have any predicted RNA structural features in common with one another or with the previously identified high affinity Rev binding site in HIV-1 RRE (stem-loop IIB) [10], [50]. Pairwise RNA sequence and secondary structure alignments performed using RNAstructure Dynalign [51] failed to identify significant RNA structural similarities in EIAV RBR-1 and RBR-2 (data not shown). In striking contrast, the stem-loop IIB region of HIV-1 and RBR-2 of EIAV, both of which correspond to high affinity Rev binding sites, have potential to form very similar ensembles of secondary structures (two examples are shown in Figure 8A).

The unexpected RNA secondary structural similarities detected through pairwise analysis of the Rev-binding domains in HIV-1 and EIAV prompted us to generate a computational RNA structural motif model based on shared features of the two regions using RNAMotif [52]. The resulting RNA motif model was used to scan the complete genomic sequences of 10 different lentiviruses, as well as the recently identified rabbit endogenous lentivirus type K (RELIK) (see Materials and Methods). Most of the genomes, ranging from 7732 to 10,359 nt in length, contain only 1 or 2 copies of the motif (Table S1); Figure 8B illustrates 9 examples. In all except 3 cases (Visna virus, CAEV and BIV), the RNA motif is located within the env gene. Both EIAV and HIV-1 have 2 copies of the RNA motif within the mapped RRE. In 4 other cases, a single copy of the motif occurs either within or <250 nt from the proposed boundaries of RRE regions mapped in previous studies (see Table S1). The striking conservation of this RNA structural motif, together with its occurrence in or near the known RRE regions of 4 additional lentiviruses and within the RELIK sequence, suggests that it may play an important role in Rev-RRE recognition, not only in HIV-1 and EIAV, but potentially in all lentiviruses.

## Discussion

The Rev-responsive element (RRE) is an essential *cis*-acting RNA regulatory element recognized by the Rev proteins of lentiviruses, including HIV-1 and EIAV. Rev-RRE interactions regulate viral gene expression and genomic replication by mediating the export of incompletely spliced or unspliced lentiviral mRNAs from the nucleus to the cytoplasm of infected host cells [7], [9]. In HIV-1, the RRE secondary structure has been extensively characterized [18], [19], [50], [53] and systematic functional and structural studies have been performed to investigate the detailed mechanism of HIV-1 Rev-RRE interactions [54], [55], [56], [57], [58], [59]. A high-resolution NMR structure for a Rev-RRE complex containing an HIV-1 Rev peptide (27 aa) bound to a synthetic RNA (39 nt) corresponding to its high affinity RRE binding site has been reported [20], but high resolution structures for the complete HIV-1 Rev protein or Rev-RRE complexes are still lacking. A three-dimensional model has been presented for a 56 nt RNA corresponding to HIV-1 RRE domain II [60]. Despite these extensive studies, the extent to which molecular mechanisms that regulate Rev-RRE interactions in HIV-1 are conserved among the lentiviruses is not known. Although previous computational studies identified potential conserved secondary structural elements in lentiviral genomes and RRE regions [25], no information regarding their functions was provided. Here, we provide evidence for involvement of a conserved RNA structural motif in Rev-RRE recognition. By analyzing the RNA secondary structure and Rev binding sites within the RRE of EIAV, the most divergent among known lentiviruses, we identified a RNA structural motif that co-localizes with high affinity Rev binding sites in both HIV-1 and EIAV, and is conserved in several other lentiviruses. These results provide a comparative framework for investigating critical features of Rev-RRE interaction that could be targeted in novel anti-lentiviral therapies.

### An Experimentally Validated RNA Structure for the EIAV RRE

Two important aims of this study were to determine the secondary structure of the EIAV RRE and to obtain high-resolution experimental data regarding Rev-RRE interactions, for comparison with available HIV-1 data. Using a combination of computational and experimental approaches, we generated an optimized RNA secondary structure model for the EIAV ERRE. In the context of this structure, individual ribonucleotides and secondary structural elements that are protected from chemical cleavage or undergo structural transitions upon Rev binding were identified using detailed RNA footprinting experiments.

One striking characteristic of our proposed ERRE structure is the relative paucity of stable stem structures and abundance of single-stranded loops, several of which are unusually large. The calculated free energy of the optimized ERRE secondary structure, based on a combination of thermodynamic calculations, chemical probing data and ribonucleotide covariation frequencies using RNAalifold is −77.9 kcal/mol (Figure 3), a value similar to that obtained using RNAStructure, −71.3 kcal/mol (Figure 2B). These values are significantly higher than free energies of alternative secondary structures predicted using computational methods that do not incorporate experimental constraints or available phylogenetic information, e.g., −126.05 kcal/mol, using Mfold (Figure 2A). Incorporating experimental constraints has been shown to result in significant improvement in the fidelity of RNA structure predictions for a variety of RNAs using RNAStructure software [39], [61]. Thus, we believe the structural model for the ERRE shown in Figure 2A, based solely on thermodynamic calculations, is less likely to be physiologically relevant than structures presented in Figure 2B and Figure 3, which may reflect features of the ensemble of ERRE structures that exist *in vivo.*

### Two Distinct Rev Binding Domains within the RRE of EIAV

The ERRE footprinting experiments provided a detailed view of the interaction between EIAV Rev and RBR-1, which contains the ESE region previously implicated in Rev binding. Within loop B, changes in chemical reactivities of every purine residue in PuA and several As in PuB were detected, consistent with previous studies reporting decreased Rev binding when mutations converting several GAA motifs into GCAs were introduced within PuB [29]. Similarly, EIAV Rev binding activity was virtually abolished when a GAA sequence was changed to GAU in PuB [32]. Both previous studies reported that mutations in PuA had little effect on the binding affinity of EIAV Rev, again consistent with the footprinting results reported here: only a few changes in ribonucleotide accessibilities in this region were detected upon Rev binding. In contrast, Rev binding resulted in significant changes in ribonucleotide accessibilities in a third purine-rich motif, located within loop A (Figure 3), including enhanced chemical reactivities at several purines within the loop. Thus, in RBR-1, structural changes that occur upon Rev binding include enhanced nucleotide reactivities within loop A, in addition to the previously mapped Rev-binding region in loop B.

RBR-2, a novel Rev-binding region within the ERRE, was identified as second Rev-mediated footprint, encompassing relatively long tracts of residues protected from hydroxyl radical cleavage and several individual ribonucleotides with enhanced reactivity to kethoxal and DMS. Quantitative *in vitro* binding assays demonstrated that RBR-2 is required for high affinity binding of EIAV Rev; ERRE fragments containing both RBR-1 and RBR-2 have much higher affinity for EIAV Rev than those lacking RBR-2 (Figure S1).

For RBR-2, all computational methods produced the same RNA secondary structure. The Rev-induced footprint in SL-Z (nt 439–462) in RBR-2 is especially interesting because the topology of this stem-loop structure is very similar to that of stem-loop IIB in HIV-I, which corresponds to the primary high-affinity Rev binding site in the HIV-1 RRE [10], [50]. Although the sequences of the two structures differ, they share a strong protection pattern on opposite strands in the stem, characteristic of a “5′ stagger” of 7 nucleotides. A 5′ stagger in the footprinting protection pattern on complementary strands of an RNA duplex suggests interaction of the bound protein within the major groove of the RNA (as in the interaction between ribosomal protein S7 and the 3′ domain of 16S rRNA in *E. coli.* [62]). This similarity supports the hypothesis that EIAV Rev binds within major groove of the RNA stem-loop structure, SL-Z, in RBR-2 (Figure 5C), in a manner analogous to that observed for HIV-1 peptides bound to RNA oligonucleotides corresponding to stem-loop IIB [20].

In HIV-1, an extended RRE sequence that includes structured regions surrounding the stem-loop IIB binding site has been shown to enhance Rev binding and promote its multimerization on HIV-1 RNAs [19], [63]. We have occasionally observed multimerization of EIAV Rev on fragments of ERRE using *in vitro* binding assays such as UV-crosslinking and EMSA (unpublished data), but we are not aware of any direct evidence for a functional role of EIAV Rev multimerization *in vivo.* Further investigation will be required to dissect the functional relationships among RBR-1, RBR-2 and the full length EIAV RRE.

### A Conserved RNA Structural Motif for Rev Recognition in both EIAV and HIV-1

To investigate whether the RBR-2 domain might have RNA structural features in common with the high affinity Rev binding stem-loop IIB region of HIV-1, we performed pairwise comparisons using Dynalign [51]. We found that the two RRE regions can form very similar ensembles of secondary structures (Figure 8A and data not shown). Because these predictions were generated using fragments of the two RRE sequences, we tested whether such similar structures could be detected in the context of the complete genomic RNA sequences of the two lentiviruses. Using a computational RNA motif model based on common structural elements identified in the HIV-1 and EIAV Rev binding sites, a shared RNA motif was detected within the RREs of HIV-1 and EIAV. Strikingly, the same motif was found within or very near the proposed RREs in six out of ten lentiviruses examined, suggesting that it could play a role in Rev-RRE recognition in diverse lentiviruses (Figure 8). Results of our detailed footprinting experiments indicate that EIAV Rev binds in the major groove of the double-stranded RNA stem corresponding to the high affinity Rev binding site in the ERRE, as is the case for HIV-1 Rev binding to stem-loop IIB, its cognate RRE. This unanticipated similarity in the binding mode of HIV-1 and EIAV Rev supports the hypothesis that this RNA motif is a conserved feature of lentiviral Rev-RRE recognition. Future work will be directed at testing this hypothesis, through a detailed functional characterization of the RBR-2 region in EIAV. Systematic studies using both *in vitro* and *in vivo* assays to assess the functional effects of mutations that alter the sequence, but preserve (or destroy) the conserved RNA structural motif, will be required to fully evaluate its potential biological significance.

EIAV is genetically the simplest of the exogenous lentiviruses, has the smallest number of genes, and lacks a *vif* protein [64], [65]. Recently, the first endogenous lentiviruses were discovered and characterized by Katzourakik et al. [66]. Although these endogenous rabbit viruses (RELIKs) are more than 7 million years old, they have retained many lentiviral genomic structural features as well as genes for regulatory proteins such as *tat* and *rev*. Interestingly, we found the conserved RNA structural motif in RELIK sequence, also within the *env* gene region (Figure 8B). Phlyogenetic analyses suggest that, among sequences of exogenous lentiviruses, the EIAV sequence is closest to the ancient RELIK sequence [66]. EIAV therefore offers a singular opportunity for comparative analysis of molecular interactions that regulate lentiviral gene expression and may identify highly conserved interactions that could be targeted in novel anti-lentiviral therapies.

## Materials and Methods

### Preparation of ERRE RNA and purified EIAV Rev protein

The EIAV ERRE was amplified by PCR from pERRE-All [29], [34], [65] using a 5′ primer containing a T7 promoter site. The PCR product was purified using QIAquick PCR purification columns (QIAGEN, Valencia, CA), and RNA was generated by *in vitro* transcription (T7-MEGAscript; Ambion, Austin, TX). Transcribed RNA was purified using MEGAClear kits (Ambion, Austin, TX), denatured at 90°C for 2 min and annealed by slow cooling. Ethanol precipitation was performed to remove salts and concentrate ERRE RNA. Concentrated RNA was stored at −80°C. EIAV Rev protein was expressed as an MBP-ERev fusion protein and purified as described previously [27].

### Chemical probing of RRE RNA secondary structure and footprinting Rev-RRE complexes

Prior to chemical probing or footprinting experiments, the annealed ERRE in RNA storage buffer (10 mM Tris-HCl, pH 7.5) was pre-incubated at 42°C for 15 min. To generate unmodified and modified unbound ERRE samples for RNA secondary structure probing experiments, two aliquots (each containing 35 pmol) of ERRE were added to RNA binding buffer (10 mM HEPES-KOH, pH 7.5, 100 mM KCl, 1 mM MgCl_2_, and 0.5 mM EDTA). To generate ERev-ERRE protein-RNA complexes for footprinting experiments, samples containing one to thirty-fold molar excess of purified MBP-ERev fusion protein were incubated with pre-folded ERRE RNA (35pmol) in binding buffer in a total volume of 87.5 µℓ for 20 min on ice. Samples containing folded ERRE RNA alone or RNA-protein complexes were incubated with 10.5 µℓ 880 mM dimethylsulfate (DMS) (Sigma-Aldrich, St. Louis, MO) or 7 µℓ 720 mM kethoxal (ICN, Costa Mesa, CA) or 16 µℓ hydroxyl radical probing mixture (4 µℓ of 50 mM Fe(NH_4_)_2_(SO_4_)_2_·6H_2_O, 4 µℓ of 100 mM EDTA, 4 µℓ of 250 mM ascorbic acid, and 4 µℓ of 2.5% hydrogen peroxide) for 10 min at room temperature. DMS modification reactions were stopped by addition of 59.3 µℓ of DMS stop buffer (1 M Tris-HCl pH 7.5, 0.1 M EDTA pH 8.0, and 1 M 2-mercaptoethanol). For the kethoxal probing, 8.2 µℓ of 0.5 M potassium borate was added for stabilizing kethoxal. Hydroxyl radical probing reactions were quenched by the addition of 92.8 µℓ of 1 M thiourea. After ethanol precipitation of RNA or RNA-protein complexes, 3 phenol and 2 chloroform extractions were performed to purify RNA from RNA-protein complexes. RNA was concentrated and washed using 100% and 70% ethanol precipitations. Finally, RNA was resuspended in 35 µℓ of water (for DMS and hydroxyl radical modifications) or 35 µℓ of 40 mM potassium borate for kethoxal modification. Additional details are provided in [43].

### Primer extension analysis of chemically-modified EIAV RRE sequences

To identify positions of chemically modified nucleotides in ERRE, primer extension analysis was used using 5 different oligonucleotide primers (Table 1), essentially as previously described in [43], [67].

### Nitrocellulose filter binding assays

Nitrocellulose filter binding assays were carried out using purified ^32^P-labelled RNAs corresponding to the ERRE (ERRE_RBR12, 555 nt; 5280 to 5834) or subfragment of the ERRE (ERRE_RBR1, 123 nt; 5443 to 5465) using standard procedures. Binding affinities were calculated using Graphpad Prism 5 software [68].

### Sequences of gp90 (SU) variants and information content analysis

The EIAV genomic RNA sequences used in the present study were originally collected for analyzing sequence variants in the EIAV Env SU protein by Mealey, et al. [42] and deposited in the NCBI GenBank in two segments (5′ and 3′). From a total of 284 EIAV sequences used in Mealey, et al., those that overlapped the ERRE region were collected. After removal of sequences with deletions or premature stop codons, a total of 258 sequences (126 corresponding to the 5′ fragment and 132 corresponding to the 3′ fragment of SU) corresponding to 139 complete *env* gene variant sequences, remained. The concept and methods for analysis of information content are described elsewhere [46], [69], [70]. Briefly, collections of the 5′ or 3′ fragment sequences were aligned using CLUSTALW (http://www.ebi.ac.uk/clustalw/) [71] and information content was calculated according to the following equation:
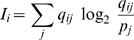
where *I_i_* is the information content for the nucleotide position *i* in the alignment, the index *j* sums over all possible nucleotides (A, T, G, and C), *q_ij_* represents the observed frequency of nucleotide *j* at position *i* and *p_j_* represents the expected frequency value, which is 0.25. The calculated information content at each nucleotide position was plotted using Microsoft Excel.

### RNA secondary structure prediction

Different methods for RNA secondary structure prediction have been reviewed recently [72], [73]. In this work, Mfold [36] was used to predict the lowest free energy secondary structure of the ERRE, using only the standard EIAV sequence as input (i.e., with no experimental constraints). To model the secondary structure of the ERRE, using a single sequence of the ERRE and experimental constraints derived from the results of chemical probing experiments, four different methods were used: Mfold (http://frontend.bioinfo.rpi.edu/applications/mfold/cgi-bin/rna-form1.cgi) [36], Sfold (http://sfold.wadsworth.org) [38], RNAfold (http://www.tbi.univie.ac.at/ivo/RNA/) [40], and RNAStructure (http://rna.urmc.rochester.edu/rnastructure.html) [39]. Experimental constraint data were preprocessed to generate appropriately formatted input files for each of the four programs. A fifth method, RNAalifold, generates an optimal RNA secondary structure based on calculation of the minimum free energy structure, and a partition function and base-pairing probability matrix derived from a multiple sequence alignment (http://www.tbi.univie.ac.at/ivo/RNA/) [41]. Input for RNAalifold consisted of an alignment of 140 ERRE variant sequences generated using CLUSTALW (http://www.ebi.ac.uk/clustalw/) [71]. The resulting alignment and experimental constraint input was used to determine an optimal RNA secondary structure for the ERRE. PSEUDOVIEWER2 (http://wilab.inha.ac.kr/pseudoviewer2/) [74] was used to generate RNA secondary structure diagrams.

### Identification of a conserved RNA structural motif within lentiviral genomes

RNAstructure Dynalign software (http://rna.urmc.rochester.edu/rnastructure.html) [51] was used to test whether sequences corresponding to the high affinity Rev binding sites of EIAV and HIV-1 have the capacity to form similar RNA secondary structures. Sequences of 100 nt regions encompassing the HIV-1 stem-loop IIB region (nt 8–107) and the EIAV RBR-2 region (nt 371–470) were compared, resulting in two very similar ensembles of predicted secondary structures. RNA motif models based on common features of the HIV-1 and EIAV RRE structures were generated using RNAMotif (http://www.scripps.edu/mb/case/casegr-sh-3.5.html) [52], and used to scan the complete genomic sequences of ten different lentiviruses (GenBank accession no. M15654;HIV-1, NC_001450; EIAV, NC_001722; HIV-2, NC_001549; SIV, NC_001452; VV, NC_001463; CAEV, NC_001511; OLV, NC_001482; FIV, NC_001413; BIV, NC_001654; JDV) and rabbit endogenous lentivirus type K (RELIK) [66].

## Supporting Information

Table S1 Occurrence of conserved RNA structural motifs in lentiviral genomes.
(0.02 MB XLS)

Figure S1 Deletion of RBR-2 from EIAV RRE results in loss of high affinity Rev binding *in vitro*. Quantitative nitrocellulose filter binding assays were performed to measure binding affinity of RNAs that contain both RBR-1 and RBR-2 (ERRE_RBR12, blue) and those that contain only RBR-1 (ERRE_RBR1, red). Purified ^32^P-labeled RNAs were incubated with increasing concentrations of purified MBP-ERev, and RNA-protein complexes specifically bound to nitrocellulose were separated from unbound RNAs and quantified by scintillation counter. Binding isotherms based on experimental data were fit to models generated using Prism software (http://www.graphpad.com/prism/Prism.htm). Binding constants estimated from these models were: K_d_ = 19 nM for RNA containing both RBR- and RBR-2 (blue) and K_d_ = 5200 nM (5.2 µM) for RNA lacking RBR-2 (red).
(0.17 MB TIF)
